# Determinism in Cyber-Physical Systems Specified by Interpreted Petri Nets

**DOI:** 10.3390/s20195565

**Published:** 2020-09-28

**Authors:** Remigiusz Wisniewski, Iwona Grobelna, Andrei Karatkevich

**Affiliations:** 1 Institute of Automatics, Electronics and Electrical Engineering, University of Zielona Góra, 65-417 Zielona Góra, Poland; i.grobelna@iee.uz.zgora.pl; 2 Department of Applied Computer Science, AGH University of Science and Technology, 30-059 Kraków, Poland; karatkevich@agh.edu.pl

**Keywords:** Petri nets, determinism, control logic, control systems, cyber-physical systems

## Abstract

In this paper, we study selected aspects of determinism in the control part of a cyber-physical system (CPS) that is specified by a Petri net-based model. In particular, the control interpreted Petri nets (CIPNs) are applied, which are an extension of the ordinary Petri nets, supplemented by signals (related to sensors and actuators) that permit communication with the environment. The notions of weak and strong determinism in a system described by a CIPN are introduced in the paper. The proposed concepts are supported by formal definitions and theorems. Moreover, a novel modelling methodology for a deterministic system specified by a CIPN is proposed. The presented solutions are illustrated by a case study example of a real-life cyber-physical system. Finally, the results of experimental verification of the proposed determinism-based techniques are demonstrated and discussed.

## 1. Introduction

A cyber-physical system (CPS) is an integration of computation with physical processes. Its behaviour is specified by the deeply intertwined software and physical components of the system [1]. The design methodology of such systems includes the joint dynamics of computers, software, networks, and physical processes. Cyber-physical systems are used in a variety of domains, e.g., transport, health care, smart homes, social networks, power management, data centres, energy systems and networking systems [2,3,4,5,6,7,8,9,10,11,12,13].

The physical part of the CPS refers to the real world and is prone to some environmental influences, while the control (software) part manages the physical objects and makes necessary decisions. Hence, the main purpose of the control part of the CPS is to monitor the environment and to control specific objects. An image of the world is created by the use of multiple input signals, coming from various sensors or coexisting systems, such as a computer vision system. The influence on the outside world is affected by various output signals, which can control the executive elements or become input signals for other parts of a CPS.

The development process of the CPS involves three major parts, namely modelling, design and analysis [1]. Modelling is defined as the understanding of a system through imitation. Models reflect system properties and specify what a system does. Whenever possible, models should preserve determinism [14], which has been proved to be extremely valuable and practical. Design is understood as a structured creation of the artefacts and specifies how a system does what it is supposed to do. The last stage, analysis, is described as gaining a deeper understanding of a system through an examination of the interactions between its components and making sure that a system does what the model specifies.

The specification phase of the CPS design is especially important, as it has an influence on further stages of development, in particular on forming a prototype of the system. It is also essential that the specification of the CPS should be supported by formal verification at all project stages. According to a report from the beginning of the CPS era [15], there was a need for research in the CPS domain. New models, algorithms, methods and tools were necessary to incorporate the verification and validation of software and systems at the control stage of design. Additionally, in [16], it was concluded that existing computing and networking abstractions had to be rebuilt in order to use the full potential of CPSs. Since that time, a large amount of valuable research has been conducted, some of which will be briefly presented below.

The various specification and development methods of CPS can be found in the literature. In [17], a framework for developing a CPS is proposed, based on a model-driven approach with quality assurance and high-level Petri nets. Modelling with high-level Petri nets is discussed in more detail in [18], where several patterns for modelling various behavioural features are provided. Hybrid Predicate Transition Nets (HPrTNs), related to hybrid automata, for the modelling and analysing of CPSs are introduced in [19]. In [20], Petri nets are applied for the specification of a CPS dedicated to the control of a direct matrix converter with space vector modulation and transistor commutation [21]. A model-integrated development approach through a wide use of models is introduced in [22], covering all aspects of the hardware and software components and their interactions. To specify hardware platform and software components, the EsMoL modelling language is used. A Domain Specific Modelling Language (DSML) for cyber-physical systems is proposed in [23], which allows the modelling of structural and behavioural aspects in a single model, but unfortunately with no design verification. Petri nets for cyber-physical system specification, combined with dataflows, are proposed in [24], offering support for the design of mixed systems with linear control and signal processing operations, as well as with event driven elements. In [25], the current state of the art of model-based methodologies for CPS are presented as a basis for future extensions of the SysML standard to support CPS modelling. The multiplicity and variety of approaches shown in the literature reveal that the topic is still relevant and that there is still much work to be done.

This paper focuses on the specification aspects of the control part of a CPS specified by a Petri net. The most popular modelling methods of CPS described by Petri nets are based on the application of specialized net classes and their extensions [26] or concentrate on a specific aspect (e.g., system security [27]). In this work, a more general approach is proposed. The presented idea involves interpreted Petri nets, which take into account the input (e.g., sensors) and output (e.g., actuators) signals of the system. Such signals allow for bidirectional communication with the physical world. They are usually employed to control other components of the system.

Going into more detail, this paper pays special attention to the deterministic aspects of the CPS specified by an interpreted Petri net. Such a net allows an easy modelling of a concurrency in the prototyped CPS, and this is an obvious advantage. However, parallelism may also lead to an undetermined behaviour of the system. The well-known notion of determinism of the sequential finite state machines (see Definition 12 in Section 2) is evidently inapplicable to the parallel systems directly. In such systems, under some circumstances, two or more transitions may fire at the same time [28], hence, the requirement that “for each state there is at most one transition enabled by each input value” does not hold anymore [1]. This means that for the modelling of concurrent control processes a slightly different definition of determinism in the interpreted Petri nets is needed.

Furthermore, according to [29] “determinism” is a property of a model (not of a physical realization). The nondeterminism itself can be handled using, e.g., actors in programming languages and frameworks with parallel and distributed computing dataflow dialects, process networks, synchronous-reactive models or discrete-event models [30]. Indeed, the idea proposed in this paper is close to the synchronous-reactive (SR) principle from [30], where transitions (originally the actors) react simultaneously and instantaneously at each tick of a global clock, which is valuable in the case of many cyber-physical systems. However, unlike in [31], we do not use logical timestamps to ensure the determinism.

Finally, it should be mentioned that researchers familiar with Petri net theory have already dealt with the determinism in a modelled system, and a lot of Petri net-based specifications use additional time variables. The examples are, among others, Deterministic Timed Petri Nets (DTPNs) [32], which introduce deterministic time delays into the net structure, or Discrete Deterministic and Stochastic Petri Nets (DDSPNs) [33], where transitions can fire either in zero time or according to arbitrary firing times (represented as time to absorption in a finite absorbing discrete-time Markov chain). Unlike the time-related approaches, we do not introduce any additional timing aspects into the model.

Summarizing the above discussion, it can be noticed that the current modelling methods of CPSs specified by Petri nets have several gaps, especially in regard to the determinism of the system. In this work, we propose a novel modelling approach. The main idea is based on the application of control signals (they can also be associated with the sensors and actuators) that allow the maintenance of an on-going interaction with the remaining parts of the CPS. Furthermore, a new determinism-oriented modelling technique for a system specified by an interpreted Petri net is proposed.

The main contributions of the paper can be emphasized as follows:

- Definition of *weak determinism* in a control interpreted Petri net.
- Definition of *strong determinism* in a control interpreted Petri net.
- Proposition of three novel theorems in regard to weak and strong determinism in a system specified by a control interpreted Petri net.
- Proposition of a novel modelling methodology for the control part of the CPS. The idea is mainly focused on the determinism and verification aspects in the modelled system.
- The survey on existing approaches to determinism in Petri nets.
- Introduction of a hierarchical control interpreted Petri net (HCIPN), related to the proposed deterministic-based modelling technique.
- A case study example of a real-life cyber-physical system modelled according to the ideas and techniques proposed in this work.
- Experimental verification of the proposed determinism-based techniques.

The rest of the paper is structured as follows. Section 2 presents some necessary preliminaries. Section 3 discusses the existing approaches to determinism in Petri nets, concerning both sequential and concurrent processes. Section 4 introduces the weak and strong determinism of control interpreted Petri nets. Section 5 presents a novel modelling methodology with Petri nets focused on determinism. Section 6 illustrates the methodology. Section 7 provides experimental verification. Finally, Section 8 concludes the paper.

## 2. Preliminaries

A Petri net is a formal mathematical apparatus [34,35,36,37,38,39,40] that allows the graphical representation of a control system, with a wide support for analysis [41,42,43] and verification [44,45]. Its main elements involve places, transitions and arcs, while the current state of the system is indicated by a token or multiple tokens. Its main advantage lies in the natural reflection of the concurrency relations in the modelled system, which is essential in designing a system to be implemented on digital circuits, such as Field Programmable Gate Array (FPGA) devices [46,47]. Moreover, it can be decomposed (if needed) into smaller parts (modules), and each one can then be implemented in a separate device [20,47]. Formally, a Petri net can be defined as follows.

**Definition** **1.** *Petri net:* *A Petri net is a four-tuple* $PN=\left(P,T,F,M_{0}\right)$ *, where* P *is a finite set of places,* T *is a finite set of transitions,* F⊆(P x T) ∪ (T x P) *is a finite set of arcs,* $M_{0}$ *is an initial marking.*

Sets of input and output places of a transition are defined as: •t={p∈P: (p, t)∈F}, t•={p∈P: (t,p)∈F}; sets of input and output transitions of a place are in turn denoted as: •p={t∈T:(t,p)∈F}, p•={t∈T:(p,t)∈F}.

**Definition** **2.** *Marking: a marking (state)* M *of a Petri net* $N=\left(P,T,F,M_{0}\right)$ *is defined as a subset of its places:* M⊂P *. The set of all possible (reachable) markings is denoted by* ℳ *.*

A place belonging to a marking is called a *marked place*. A marked place contains a token. M(p)=1 if p contains a token in M (i.e., p∈M), otherwise M(p)=0.

**Definition** **3.** *Enabled transition: a transition is enabled in marking* M *iff* •t⊆M *; otherwise it is disabled.*

**Definition** **4.** *Transition firing in a Petri net: a transition of a Petri net can be fired if and only if it is enabled. The firing of a transition removes a token from each of its input places and adds a token to each of its output places, which can be described as:* $M^{\prime }=\left(M\backslash •t\right)\cup t•\;$ *(or simply:* $M\overset{t}{\to }M^{\prime }$ *).*

A marking can be changed only by the firing of an enabled transition.

**Definition** **5.** *Firing sequence: a firing sequence* $\sigma =t_{1}t_{2}\cdots t_{n}$ *is a sequence of transitions such that* $M\overset{t_{1}}{\to }M_{2}$ *,* $M_{2}\overset{t_{2}}{\to }M_{3}$ *, …,* $M_{n}\overset{t_{n}}{\to }M^{\prime }$ *. Then* $M\sigma M^{\prime }$ *. A marking* $M^{\prime }$ *is reachable from marking* M *if it can be reached from* M *by a sequence of transition firings.*

**Definition** **6.** *Parallel transitions: transitions* $t_{1}$ *,* $t_{2}$ *are parallel if there is marking* M∈ℳ *such that both* $t_{1}$ *and* $t_{2}$ *are enabled in* M *and* $•t_{1}\cap •t_{2}=\emptyset$ *.*

**Definition** **7.** *Liveness: a Petri net is live if from any reachable marking it is possible to fire any transition by a sequence of firings of other transitions.*

Informally, liveness means that a) no transition is initially dead and b) no transition can become dead; i.e., no transition can ever lose the possibility to fire in the future. Both conditions are important for the nets describing control systems, because a transition which is initially dead is redundant, while a transition which eventually becomes dead means that the system cannot properly function in a cyclic way, whereas cyclic behaviour is typical for the control systems.

**Definition** **8.** *Safeness: a place of a Petri net is safe if:*

∀M∈ℳ,∀t∈T,∀p∈P:((•t⊆M)∧(p∈t•)⇒(p∉M)∨(p∈•t)).

Informally, safeness means here that no more than one token can appear in a place. It is typical for the interpreted Petri nets (including the model defined below) that some actions are performed, when a place has a token, but it is not specified what happens when it has more than one token.

In a general sense, liveness means that “something good will eventually occur”, when safeness (or safety) means that “something bad will never happen” [48]. Applying these properties to the classical Petri nets, they are usually narrowed as described in Definitions 7 and 8. Because of the importance of the mentioned properties, a live and safe Petri net is called a well-formed net [39].

Interpreted Petri nets are usually employed to describe the real-life systems, such as concurrent controllers. A control system communicates with the environment by means of input and output signals (ports). The input signals or their combinations specify the guards of transitions, while outputs are bound to the places of the net. Various definitions of interpreted Petri nets can be found in the literature. In some cases, the nets are narrowed to be live and safe [34,49], and sometimes there are no such severe restrictions [50,51]. Such a lack of standardization can lead to confusions. Therefore, to be clear and unambiguous, in our approach we decided to use live and safe nets. Formally, an interpreted Petri net can be defined as follows.

**Definition** **9.** *Control interpreted Petri net: a control interpreted Petri net (CIPN) is a live and safe Petri net, defined as a six-tuple:* $IPN=\left(P,T,F,M_{0},X,Y\right),$ *where* X *is a finite set of binary inputs and* Y *is a finite set of binary outputs,* X∩Y=∅ *. A guard, as a Boolean function of the inputs, may be associated with a transition;* g(t,x) *means the value of guard of transition* t *when the input values are* x *(lack of the guard is considered as constant 1). Function* $\lambda :P\to 2^{Y}$ *associates a subset of* Y *(maybe empty) to every place. An output* y∈Y *has a value of 1 at marking* M *if and only if there is a marked place* p *with which it is associated:* (∃p∈P: y∈λ(p),M(p) = 1) ⇒y = 1 *.*

By x we mean a Boolean vector representing the values of the input signals.

**Definition** **10.** *Transition firing in a CIPN: a transition of a CIPN is fired immediately when and only when it is enabled, and its guard is fulfilled. The firing of a transition changes the marking in the same way as it is described in Definition 4. If there are two or more enabled transitions with fulfilled guards sharing the same input place, at most one of them is fired nondeterministically.*

**Definition** **11.** *Stable marking: a marking* M *of a CIPN is stable, with regard to **x**, if for any transition* t *enabled in* M *:* g(t,x)=0 *.*

The stability of a marking means that the marking M is not going to change until the input remains unchanged (no transition can fire because for any enabled transition its guard is not satisfied).

Note that there are different models of interpreted Petri nets. For example, in some of them, the firing of a transition can assign a value of 0 or 1 to an output signal, or X∩Y≠∅ [41,52]. However, the model used in this paper is defined by Definition 9.

Finally, let us recall the definitions of determinism in a state machine and generally in a model. These notions will be a starting point for our further considerations.

**Definition** **12.** *Determinism in a state machine: a state machine is deterministic if, for each state, there is at most one transition enabled by each input value.*

**Definition** **13.** *Determinism in a model: a model is deterministic if it is given an initial state of the model and all the inputs that are provided to the model, and the model defines exactly one possible behaviour. In other words, a model is deterministic if it is not possible for it to react in two or more ways to the same conditions [29].*

When designing the control part of the CPS, it is sometimes necessary to use smaller modules within the specification and to embed hierarchy in the model. Firstly, it increases the readability so that a specification at the highest level of abstraction, including multiple modules, is still clear enough to understand. Secondly, the modules consisting of a relatively limited number of places and transitions are easier to analyse and to verify (in comparison to a single flat Petri net model). Thirdly, the conception of modules allows a better modelling of the determinism. Taking into account the fact that several modules may be active at the same time (which is obviously a desired behaviour of the net) and that we are not trying to interfere in the order of transition firings within the modules, the parallel modules should behave independently of each other. Additionally, we assume that the modules do not communicate with each other via the signals. Any needed synchronization or communication is realized at a higher level by means of structural mechanisms (places and transitions). There exist different models of hierarchical Petri nets, sometimes containing a lot of elements which are not necessary for our modelling purposes (e.g., [44,53,54,55,56]). 

Below we formally define a module and a hierarchical control interpreted Petri net, adequate for the considered task.

**Definition** **14.** *Module CIPN: a module CIPN (MCIPN) (module for short) is a control interpreted Petri net*$MCIPN=\left(P,T,F,p_{in},p_{out},X,Y\right)$*such that P,T,F,X,Y have the same meaning as in Definition 9; transition firing occurs according to Definition 10;*$\exists p_{in}\in P:\;•p_{in}=\emptyset$*;*$\exists p_{out}\in P:\;p_{out}•=\emptyset$*;*$\forall p\in P\backslash \left\{p_{in},p_{out}\right\}:\left(•p\neq \emptyset \right)\;\wedge \left(p•\neq \emptyset \right)$*. Places*$p_{in}$*and*$p_{out}$*are called the initial place and the terminal place of the module, respectively.*$M_{in}=\{p_{in}\}$*. From any marking*M*which is reachable from*$M_{in}$*, the terminal marking*$M_{out}=\{p_{out}\}$*is reachable, and no marking*$M^{\prime }$*is reachable such that*$M^{\prime }⊃M_{out}$*. MCIPN with added transition*$t^{\prime }$*such that,*$•t^{\prime }=\left\{p_{out}\right\}$, $t^{\prime }•=\left\{p_{in}\right\}\;$*is live, considering*$M_{0}=M_{in}$.

**Definition** **15.** *Hierarchical control interpreted Petri net: a hierarchical control interpreted net Petri net (HCIPN) is a* $CIPN=\left(P,T,F,M_{0},X,Y,\right)$ *such that some of its places* $P_{m}\subseteq P$ *are the macroplaces. **χ** is a hierarchy function which associates a module CIPN with every macroplace* $p_{m}$ *. Adding a token to macroplace* $p_{m}$ *adds a token to the initial place* $p_{in}$ *of the module* $MN=\chi \left(p_{m}\right)$ *. If* $p_{m}\in M_{0}$ *, then in the corresponding module* $MN=\chi \left(p_{m}\right)$ *its input place* $p_{in}$ *initially has a token, and all other places of the modules are initially empty. Any transition* $t\in p_{m}•$ *can be enabled only when the output place* $p_{out}$ *of the corresponding module is marked. Removing a token from* $p_{m}$ *by the firing of transition* t *also removes the token from* $p_{out}$ *. The highest level of HCIPN (the net which is not associated with a macroplace) is called a top module.*

A top module, unlike all the other modules, does not have input and output places.

Note that in this paper we consider the modules as the nets which can also contain macroplaces, according to the definition of HCIPN. This means that the hierarchical CIPNs can be multilevel.

## 3. The Survey on Existing Approaches to Determinism in Petri Nets

Classical Petri nets [41] are not deterministic. When transition t is enabled, it can fire, but the model does not specify when the firing happens (or whether it is going to happen at all, if the firing of another transition can remove the tokens from the input places of transition t). Of course such a highly abstract model is useful for modelling in many cases, but it is not enough for the specification of a real live system such as a control system. This is the reason why the different variants of the interpreted Petri nets were developed. In such models [52,53,54,55,57,58], the guards are added to the transitions. These guards are the Boolean functions depending on the input signals. Besides, it is assumed that an enabled transition fires immediately when its guard returns to a value of 1. This changes a nondeterministic Petri net into a deterministic model. Of course, there are differences between synchronous and asynchronous interpretations (in the first case, all transitions an enabled such that their guards allow firing, and they fire in the nearest clock cycle [59]; in the second case, an enabled transition fires immediately when the guard is satisfied [60]).

This is not enough to guarantee the deterministic behaviour; the guards should also be able to resolve the conflicts, which means that for any two transitions which have common input places and can be enabled in the same reachable marking, the conditions of their firing should contradict each other, i.e., can never be satisfied simultaneously (though it is possible that two transitions having common input places are not enabled simultaneously in any reachable marking because of the net structure, checking whether it is so requires reachability analysis of the net). If this is the case (and if only the input signals are the arguments of the guards), then the firing of transitions is determined unambiguously by the current state and input signals. This condition is a generalization of determinism in a state machine (Definition 12).

**Figure 1:**
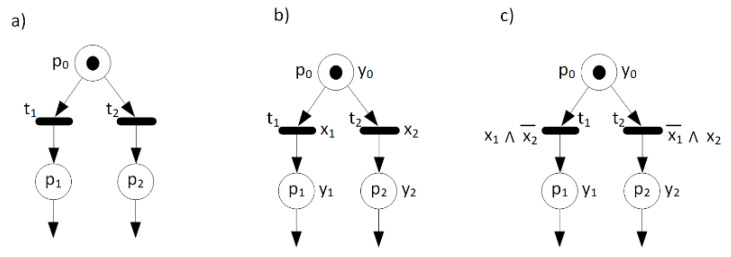
A sample Petri net without guarded transitions (**a**), with guarded transitions (**b**) and with mutually exclusive guarded transitions (**c**).

A fragment of a sample Petri net with three places and two transitions is shown in Figure 1a. Here, whenever place $p_{0}$ is marked then both transitions $t_{1}$ and $t_{2}$ are enabled. As far as we do not know which transition will fire, the system specified by this particular net is not deterministic. The conflict can be easily resolved by adding guards to the transitions. The Petri net in Figure 1b has input and output signals, but without additional assumptions the system specified by this net is still not deterministic, as far as the guards are satisfied simultaneously, when $x_{1}=x_{2}=1$. In this example, both input signals (being guards for transitions *t_1_* and *t_2_*) ought to be mutually exclusive and the following condition ought to be fulfilled: $x_{1}x_{2}\equiv 0$. Modification of the guards such as shown in Figure 1c can guarantee such a mutual exclusion.

An alternative way of solving the conflicts in a deterministic way is introducing the priorities. In the priority nets, when two or more conflicting transitions are simultaneously enabled, the transition with higher priority fires [61]. In many cases priorities can be established with the help of the guards [62].

Another aspect of providing determinism in the concurrent systems is related to the notion of the diamond rule [63]. The diamond rule means the commutativity of enabled independent transitions, i.e., the state of the system after the firing of two (or more) concurrently enabled transitions does not depend on their firing order. This property always holds for the classical Petri nets, but for many of their extensions, such as interpreted Petri nets, some additional conditions may be required, and holding of this rule is necessary for the deterministic behaviour of the systems. For some variants of the interpreted Petri nets this is related to the assignment of values to the outputs or the internal variables.

Two ways of such an assignment (or their combination) are possible: when the values of those variables are unambiguously defined by the current marking [47] or when the values are assigned if a transition is fired [52]. In the second case, determinism requires that the concurrent transitions should never try to assign different values to the same variable. In [52], such a property is called consistency. The situation becomes even more complicated in the case of asynchronous interpretation and presence of the internal variables participating in the transition guards, such that other transitions assign values to them. Then it is necessary for a deterministic model that if two transitions are parallel, the firing of one of them does not violate the condition of the firing of the other. In [52], such a property is called persistency. Verifying whether an interpreted Petri net is consistent and persistent requires knowing whether a parallelism relation between the transitions is present.

For the control interpreted Petri nets model considered in this paper, consistency and persistency are always satisfied, because, according to Definition 9, values of the output signals depend on the current marking only, and the output signals cannot be the arguments of the guards.

Another approach ensuring determinism in the Petri net model is the concept of time intervals and time stamps [64]. In timed Petri nets “a transition with firing capability may fire only during the given interval and must have fired at the latest by the end of the interval, except when it loses its firing capability in the meantime” [56]. If such an interval is narrowed specifying a concrete delay of a transition firing, the model becomes deterministic. Time dependencies are extremely important in the design and analysis of the control systems; however, they are beyond the scope of this paper.

There are models combining different approaches to provide determinism. For example, the model used in CPN tools [65] and RTCP nets [54] include guards, priorities and time stamps.

In control systems [26,49,66], some of the functionality can be performed simultaneously in concurrent processes. Petri nets allow the modelling of concurrency, and some hardware platforms are especially suited to be applied in concurrent control systems (such as FPGA devices). The main benefit of concurrent processes is the fact that they can be executed at the same time and that it does not matter which of them finishes earlier (assuming synchronization at the end and no shared data). Hence, if we want to ensure determinism according to the classical definition of determinism in a state machine (i.e., for each state there is at most one transition enabled by each input value, see Definition 12), then the advantages of the concurrency would be lost.

**Figure 2:**
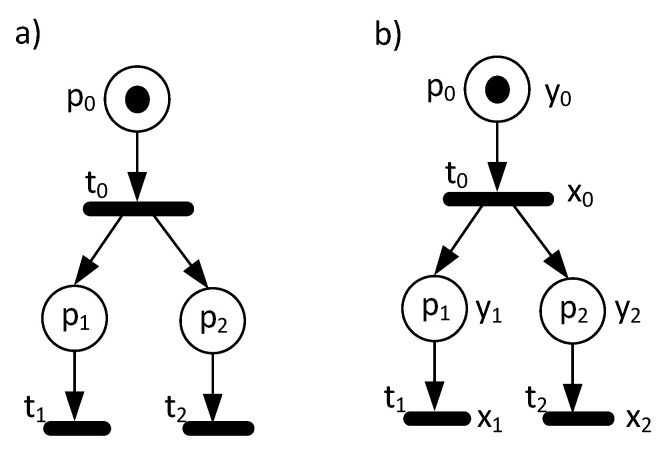
A sample Petri net with concurrent processes (**a**), and additionally with guards (**b**).

Let us illustrate it with an example. The sample Petri net in Figure 2a contains three places and three transitions. There are no conflicts in this net. However, an analysis of the behaviour reveals that once transition $t_{0}$ fires, then places $p_{1}$ and $p_{2}$ are marked simultaneously, and both transitions $t_{1}$ and $t_{2}$ are enabled. It means that we do not know which transition is going to fire first. Consider the Petri net with additional input and output signals assigned to transitions and places, shown in Figure 2b. Initially, transition $t_{0}$ is enabled and fires when $x_{0}$ becomes active. Then, places $p_{1}$ and $p_{2}$ are marked simultaneously, and the operations are denoted by $y_{1}$ and $y_{2}$. Note that the output transitions of those places ($t_{1}$, $t_{2}$) are enabled in the obtained marking. Furthermore, the order of firing strictly depends on the input signals assigned to these transitions (x, $x_{2}$). Hence, in order to provide that their firing is fully deterministic, the input signals assigned to the transitions should not obtain a value of 1 at the same time. However, this solution is needed only if we intentionally want to order all the events in the net deterministically or if the concurrent processes share some data or need to be somehow synchronized.

From the discussion above, it seems reasonable to concretize Definition 13 for CIPNs in two ways, introducing the notions of weak and strong determinism, which follows in the next section.

## 4. Weak and Strong Determinism in Control Interpreted Petri Nets

Definitions 12 and 13 lead to the further defining of determinism in an interpreted Petri net, whereby two kinds of determinism have to be distinguished: weak and strong.

**Definition** **16.** *Weak determinism in a CIPN: a control interpreted Petri net is (weakly) deterministic, if for each reachable state (marking)* M *and for any fixed input values* x *the net comes into a stable marking* M′ *and at the same time there is no stable marking* $M^{\prime \prime }\neq M^{\prime }$ *into which the net can come from* M *with the same input values.*

**Definition** **17.** *Strong determinism in a CIPN: a control interpreted Petri net is strongly deterministic if it is weakly deterministic and for each reachable marking and any fixed input values there is only one next marking possible.*

In other words, an interpreted Petri net is deterministic if it is not possible for it to react in two or more ways to the same input values in a particular state (according to the general Definition 13). However, it should be clarified which ways of reacting are understood as different ones. Informally, the difference between weak and strong determinism is that for a weakly deterministic net, for marking M and inputs x, the next stable state $M^{\prime }$ is unambiguously defined by M and ***x***, but different firing sequences and different sequences of intermediate states are possible on the way from M to $M^{\prime }$. For a strongly deterministic net, having marking M and inputs x, only one (maybe empty) sequence of the following markings is possible. It means that for a strongly deterministic net, for marking M and inputs x, the next marking $M^{\prime }$ (obtained by the firing of at most one transition) is unambiguously defined by M and x. The net shown in Figure 2a is weakly deterministic, but not strongly deterministic. The net shown in Figure 1c is strongly deterministic.

The following theorems specify conditions of weak and strong determinism in the interpreted Petri nets.

**Theorem** **1.** *A control interpreted Petri net is weakly deterministic if the next two conditions are satisfied: (1) for any two transitions with a common input place their guards never obtain a value of 1 simultaneously and (2) for each reachable marking and any fixed input values, the net eventually comes into a stable marking (an infinite looping is impossible).*

**Proof.**  If the second condition is not satisfied, then there is an evident contradiction with the definition of weak determinism. Suppose that both conditions are satisfied, and the net is not weakly deterministic. Then let M be a marking, and x be a vector of input signals. According to the second condition, the net will come to a stable marking. Let us show that there is only one such marking. Suppose the opposite: $M^{\prime }$ and $M^{\prime \prime }$ are two different stable markings into which the net can come from M with the inputs x. Let $M\sigma^{\prime }M^{\prime }$, $M\sigma^{\prime \prime }M^{\prime \prime }$. Without a loss of generality, suppose that the first transitions of $\sigma^{\prime }$ and $\sigma^{\prime \prime }$ are different ($t^{\prime }$ and $t^{\prime \prime }$, respectively). Then, $t^{\prime }$ should be disabled in $M^{\prime \prime }$ (as far as $M^{\prime \prime }$ is stable and the input signals are the same) and it cannot be disabled by firing of another transition (otherwise condition 1 is not satisfied). Then, $t^{\prime }\in \sigma^{\prime \prime }$. Analogously, $t^{\prime \prime }\in \sigma^{\prime }$. Both $t^{\prime }$ and $t^{\prime \prime }$ can fire at M, and from condition 1 $t^{\prime }$ and $t^{\prime \prime }$ have no common input places and cannot be disabled by any other transition belonging to $\sigma^{\prime }$ or $\sigma^{\prime \prime }$; neither the firing of $t^{\prime }$ or $t^{\prime \prime }$ can disable any other transition belonging to $\sigma^{\prime }$ or $\sigma^{\prime \prime }$(in the other words, $t^{\prime }$ and $t^{\prime \prime }$ are independent with respect to themselves and of any other transition $t^{\prime \prime \prime }$ enabled in M such that $g\left(t^{\prime \prime \prime },x\right)=1$). Then, both firing sequences $\sigma^{\prime }$ and $\sigma^{\prime \prime }$ can be re-ordered in such a way that $Mt^{\prime }t^{\prime \prime }M^{\prime \prime \prime }\sigma_{1}M^{\prime }$, $Mt^{\prime }t^{\prime \prime }M^{\prime \prime \prime }\sigma_{2}M^{\prime \prime }$ (from the diamond rule, such re-ordering does not change the resulting marking). Both sequences are finite, and the continuation of such reasoning will lead to the conclusion that $M^{\prime }\;=\;M^{\prime \prime }$, i.e., to a contradiction. Hence, the net is weakly deterministic. □

**Lemma** **1.** *Let*M*be a marking of a safe Petri net—*$t_{1}$*and*$t_{2}$*are the transitions such that*$•t_{1}\neq t_{1}•$*,*$•t_{2}\neq t_{2}•$*,*$t_{1}\neq t_{2}$*(which means that*$•t_{1}\neq •t_{2}$*or*$t_{1}•\neq t_{2}•$*), and both*$t_{1}$*and*$t_{2}$*are enabled in*M*. Then*$Mt_{1}M^{\prime }$*,*$Mt_{2}M^{\prime \prime }$*,*$M^{\prime }\neq M^{\prime \prime }$.

**Proof.**  From the conditions of the lemma, at least one of the places exists such that:

1. $p_{1}\in •t_{1}$, $p_{1}\in t_{1}•$, $p_{1}\in •t_{2}$, $p_{1}\notin t_{2}•$;
2. $p_{2}\in •t_{1}$, $p_{2}\in t_{1}•$, $p_{2}\notin •t_{2}$, $p_{2}\in t_{2}•$;
3. $p_{3}\in •t_{1}$, $p_{3}\notin t_{1}•$, $p_{3}\notin •t_{2}$, $p_{3}\notin t_{2}•$;
4. $p_{4}\notin •t_{1}$, $p_{4}\in t_{1}•$, $p_{4}\notin •t_{2}$, $p_{4}\notin t_{2}•$

(or one of four symmetric variants). Consider them:

1. $p_{1}\in M^{\prime }$, $p_{1}\notin M^{\prime \prime }$ ⇒ $M^{\prime }\neq M^{\prime \prime }$;
2. $p_{2}\in M$, $p_{2}\notin •t_{2}$, $p_{2}\in t_{2}•$ - according to Definition 8, the net is not safe - a contradiction;
3. $p_{3}\in M^{\prime \prime }$, $p_{1}\notin M^{\prime }$ ⇒ $M^{\prime }\neq M^{\prime \prime }$;
4. If the net is safe, then $p_{4}\notin M$, $p_{4}\in M^{\prime }$, $p_{4}\notin M^{\prime \prime }$ ⇒ $M^{\prime }\neq M^{\prime \prime }$.

**Theorem** **2.** *A control interpreted Petri net is strongly deterministic if and only if it is weakly deterministic and for any two transitions* $t_{1}$ *and* $t_{2}$ *such that* $•t_{1}\neq t_{1}•$ *,* $•t_{2}\neq t_{2}•$ *, there is marking* M∈ℳ *such that* $t_{1}$ *and* $t_{2}$ *are enabled in* M *, their guards never obtain a value of 1 simultaneously.*

**Proof.**  ⇒ If there is a reachable marking M and a vector of input signals *x* such that $t_{1}$ and $t_{2}$ are enabled at M and $g\left(t_{1},x\right)=g\left(t_{2},x\right)=1$, then $Mt_{1}M^{\prime }$, $Mt_{2}M^{\prime \prime }$, and from Lemma 1 $M^{\prime }\neq M^{\prime \prime }$. The next marking is not uniquely determined, which contradicts the definition of strong determinism.

⇐ If the net is not weakly deterministic then it is not strongly deterministic by definition. Suppose that the net is weakly but not strongly deterministic. Then it is possible that $Mt_{1}M^{\prime }$, $Mt_{2}M^{\prime \prime }$, $g\left(t_{1},x\right)\;=\;g\left(t_{2},x\right)=1$, $M^{\prime }\neq M^{\prime \prime }$. Then there are two transitions enabled in the same reachable marking such that their guards can be simultaneously satisfied. □

An important question, in the context of the proposed methodology, is how the determinism of the modules is related to the determinism of a hierarchical CIPN. The next statement provides the answer.

**Theorem** **3.** *Let* $N=\left(P,T,F,M_{0},X,Y\right)$ *be a weakly deterministic CIPN. Let* HN *be a hierarchical CIPN obtained from* N *by choosing a set of macroplaces* $P_{m}\subseteq P$ *and associating, with every macroplace* $p_{i}\in P_{m}$ *, a weakly deterministic module* $N_{i}$ *. Then* HN *is weakly deterministic.*

**Proof.**  Suppose that HN is not weakly deterministic. Then, according to the definition of weak determinism, either (1) there exists a reachable marking M and input x such that there exists an infinite firing sequence starting from M which is possible when x is unchanged, or (2) there exists reachable marking M and input x such that there are at least two stable markings $M_{1}$ and $M_{2}$, to which HN can come from M with fixed x.

(1) It follows from Definition 2 that the reachability set is finite ($\left|ℳ\right|<2^{\left|M\right|}$). If there is a possibility that no stable marking will be reached from M when the input values are x, then—taking into account that ℳ is finite—there is a firing sequence σ such that $M^{\prime }\sigma M^{\prime }$ in HN, $M^{\prime }\in ℳ$ and for every transition t in σ g(t,x)=1. Then, without loss of generality, there is such a loop in a single module $N_{i}$ or in the top module. However, every such module is weakly deterministic. According to the definition, a weakly deterministic net for every reachable marking and fixed input value comes to a stable marking. We have come to a contradiction.
(2) In the second case there exists two different firing sequences $\sigma_{1}$, $\sigma_{2}$ such that for all the transitions belonging to them their guards are satisfied at x and $M\sigma_{1}M_{1}$, $M\sigma_{2}M_{2}$ ($M_{1}\neq M_{2}$). Again, without a loss of generality there is at least one module in which such a situation takes place. However, as far as every module is weakly deterministic, there is a direct contradiction with the definition of weak determinism.

The theorem above deals with a two-level net, but it can be easily generalized by induction to show that a multilevel net consisting of the weakly deterministic modules is also weakly deterministic.

It is easy to see that a similar statement for strong determinism is not true—if all the modules are strongly deterministic, then the hierarchical CIPN can be weakly deterministic. We consider such a case as typical.

## 5. A Novel Deterministic-Based Modelling Method for a Control Part of a CPS Specified by ICPN

Let us now propose a novel modelling methodology for a control part of a cyber-physical system specified by a control interpreted Petri net. As shown in the previous sections, it is very hard (or even impossible) to ensure the complete determinism in regard to all components of the system, especially to those which are executed concurrently. Therefore, the presented technique is oriented to the relations between the blocks of the system that should be deterministic. To achieve this, the unfolding modularity approach is applied and the top-down specification approach is used. The particular single modules can be specified as either strongly or weakly deterministic. Nevertheless, it is suggested that strong determinism should be applied when it is possible, because it provides a better readability and easier verification of the modules. Theorem 2 demonstrates how to specify a strongly deterministic module. Furthermore, Theorem 3 shows that when the modules are weakly (or strongly) deterministic, the whole hierarchical CIPN is weakly deterministic, which means that to achieve the determinism of the whole system it is enough to provide it for the modules.

The proposed modelling method consists of four main steps:

1. Specification of the top module of the system by an interpreted control Petri net (it is the general description of the system, which includes the lower-level modules described as “black boxes”).
2. Unfolding of the modules with the “top-down” technique (modules can be nested and can contain other modules that should be unfolded at a subsequent modelling level).
3. Formal verification of the unfolded modules (with the “bottom-up” technique).
4. Formal verification of the top module.

**Figure 3:**
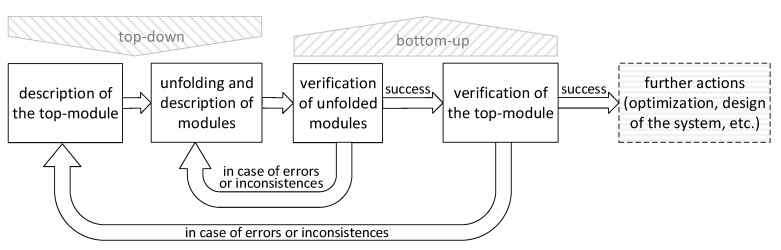
A scheme of the proposed modelling methodology.

The general scheme of the proposed modelling technique is shown in Figure 3. Let us now explain each of the above steps in more detail.

At the beginning, based on the informal sketch of the system, the general description of the modelled system is created. It contains the main blocks (called modules or macroplaces) of the system, which are treated as the black boxes with the meaning, that is not specified at that time, what happens inside a block. In the other words, each black box contains its own functionalities, which are going to be specified in the further steps of the presented method. As already mentioned, the top module is usually weakly deterministic. However, it is possible to specify it as strongly deterministic, according to the user’s needs (please refer to the next section where such a case is discussed).

The modules (macroplaces) are respectively “unfolded” (the “top-down” technique is applied). It means that the functionality of each black box is subsequently expanded. In particular, a black box may contain another nested black box (or several black boxes), which is unfolded at the subsequent level. The unfolded modules are strongly deterministic in order to ensure the proper functionality of the system, especially in regard to the order of the generated output signals.

Once all the modules are unfolded, the system is formally verified. Such an operation permits the detection of redundancy (unreachable states or transitions which can never be executed), deadlocks, and the possible re-initialization of an operation during its execution in the modelled system [41,44,47,49]. In the case of CIPNs, liveness and safeness are especially crucial properties that ought to be examined. The mentioned properties are universally important and have to be checked in virtually all cases. Although single modules (MCIPN) are by assumption not live, as they contain an initial place and a terminal place, it is still possible to check the corresponding property by joining the initial and terminal places into one place, obtaining a cyclic net. Every module corresponding to a macroplace should be live after such transformation. Together with the liveness of the top module, it provides the liveness of the whole hierarchical CIPN.

Liveness and safeness can be examined by constructing the reachability graph of a net, but there is a range of methods which allow the assessment of these properties avoiding this time- and memory-consuming approach. For example, there are reduction rules which simplify a Petri net preserving liveness and safeness properties [39]. The reduced net is usually much smaller and constructing its state space is simpler. For some classes of Petri nets, the reduction of a live and safe net leads to a trivial net with one place and one transition [52]. Safeness can be checked by means of an analysis of the covering of the net with place invariants [39]. The stubborn set approach, constructing a subgraph of the net reachability graph, finds the reachable deadlocks if they exist [67]. For some classes of nets, this approach also allows the assessment of liveness and safeness [41]. Some methods of liveness and safeness analysis are implemented in the popular PIPE tool [68]. 

Formal verification with the model checking technique allows the assessment of the specific behavioural properties (expressed with temporal logic) that take into account the input and output signals. For example, it is possible to check whether a specific state is reachable in which both signals a and b are active at the same time. This allows to check the relationships between signals.

The proposed modelling methodology applies the “bottom-up” verification technique. This means that the lower level modules are examined firstly. This process is repeated recursively, until the top-level module is verified. When the modules corresponding to the macroplaces of a CIPN are already checked, a successful verification of the net at its highest level (top-module) ensures that the whole system satisfies the requirements. If a particular model does not satisfy the requirements, appropriate counterexamples are generated [44,69]. Their analysis helps to find the possible error source. Furthermore, if some of the requirements are not satisfied in the particular modules, then the particular modules should be revised (second step) and their formal verification should be performed again (third step). If the verification of the top-module does not succeed, then the whole specification process ought to be repeated, revising all the unfolded modules (first step). 

The main advantage of the proposed modular approach relies on the examination of particular single modules in order to avoid the state explosion problem. Note that as far as a verified module is strongly deterministic, its behaviour is similar to the behaviour of a Finite State Machine (FSM), and a state explosion does not arise. Even if the modules are weakly deterministic, a hierarchical approach can reduce the number of states to be explored.

## 6. Case Study Example of the Proposed Method

**Figure 4:**
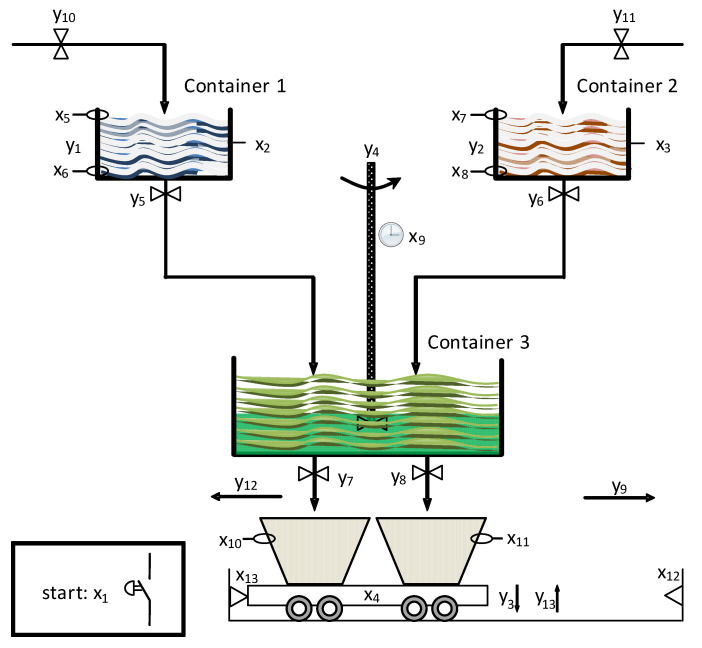
A beverage production and distribution system.

The presented method will be explained by a real-life example of a cyber-physical system. Figure 4 presents a beverage production and distribution process, initially shown in [70]. Its slightly modified version will be modelled according to the rules proposed in Section 4. 

The functionality of the system can be described as follows. At the beginning, the system remains in an idle state, until the start button (denoted by signal $x_{1}$) is pressed. It initializes the production and distribution process. Two valves (outputs $y_{10}$ and $y_{11}$) are opened and two containers are filled up by the liquid ingredients, until the proper level is achieved (signalized by sensors $x_{5}$ and $x_{7}$ for Container 1 and Container 2, respectively). Then, the ingredients are warmed up (outputs $y_{1}$ and $y_{2}$) to achieve the proper temperature. Simultaneously to the above procedure, the distribution cart is prepared. Two cups are placed on the cart (operation $y_{3}$), which is signalized by sensor $x_{4}$. Then, the cart is moved to the leftmost position (notified by sensor $x_{13}$). After that, it waits until the beverage production process is completed. 

Once the warm-up procedure is finished for both containers (sensors $x_{2}$ and $x_{3}$), their valves are opened ($y_{5}$ and $y_{6}$). The heated ingredients are poured into Container 3, where they are mixed ($y_{4}$). Such a process is executed until both containers are empty (which is signalized by sensors $x_{6}$ and $x_{8}$, respectively). Moreover, the mixing of the ingredients is controlled by a clock. Once the time is elapsed (signal $x_{9}$), the production is finished and the beverage is ready for distribution. 

The produced liquid is poured into the cups placed on the distribution cart. The filling-up process is executed independently for each cup (outputs $y_{7}$ and $y_{8}$, respectively), until the upper limit is reached (signalized by sensors $x_{10}$ and $x_{11}$). Finally, the cart transfers the beverage ($y_{9}$). It moves until reaching the rightmost position (which is notified by sensor $x_{12}$). The cups are taken from the cart ($y_{13}$), and the system is ready for further operations, awaiting the pressing of button $x_{1}$.

### 6.1. Specification of the System at a General Level (Top Module)

**Figure 5:**
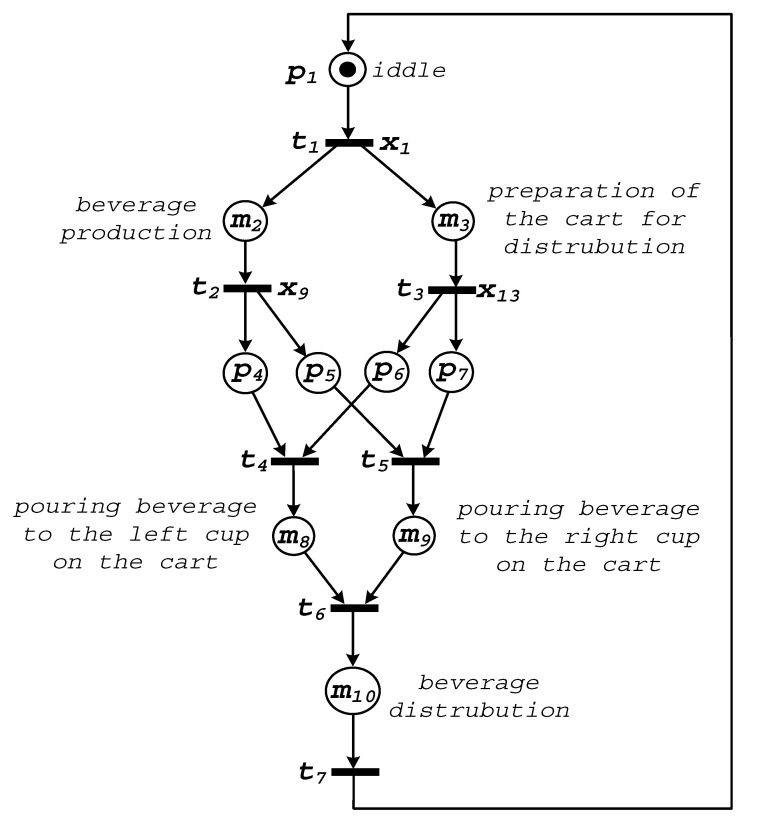
A top module of the system.

Initially, based on the informal description presented above, the system is specified at a general level. Figure 5 shows the top module of the model. There are ten places and seven transitions in the presented interpreted Petri net. Five of the places are denoted by “*m*”, since they refer to the modules that will be unfolded in further steps of the proposed method.

Let us briefly discuss the presented Petri net. The first place, denoted by $p_{1}$, refers to the idle state of the system. The pressing of button $x_{1}$ (assigned to transition $t_{1}$) starts the beverage production process (macroplace $m_{2}$), and simultaneously prepares the cart for distribution (macroplace $m_{3}$). Once the production process is finished (i.e., the time measured by a clock is elapsed and sensor $x_{9}$ is activated), transition $t_{2}$ fires and the system is ready for the distribution process. Note that this operation ought to be properly synchronized with the movement of the cart, which should be already situated at the required position (sensor $x_{13}$ tied to transition $t_{3}$). This task is solved by four synchronization places $p_{4},\ldots ,p_{7}$. Let us underline that these places are very important from the deterministic point of view, since they assure the proper sequence of generated output values (e.g., pouring of the beverage is activated only when the beverage is already produced and the cart is at the required position). Two further modules $m_{8}$ and $m_{9}$ are responsible for the simultaneous pouring of the beverage to the cups placed on the cart. Finally, the product is distributed (macroplace $m_{10}$), and the system returns to the starting position ($p_{1}$).

### 6.2. Unfolding of the System

Once the top module is specified, the system is subsequently unfolded with the usage of the top-down technique. There are five macroplaces in the top module: $m_{2}$, $m_{3}$, $m_{8}$, $m_{9}$, $m_{10}$. Let us briefly describe each of them.

**Figure 6:**
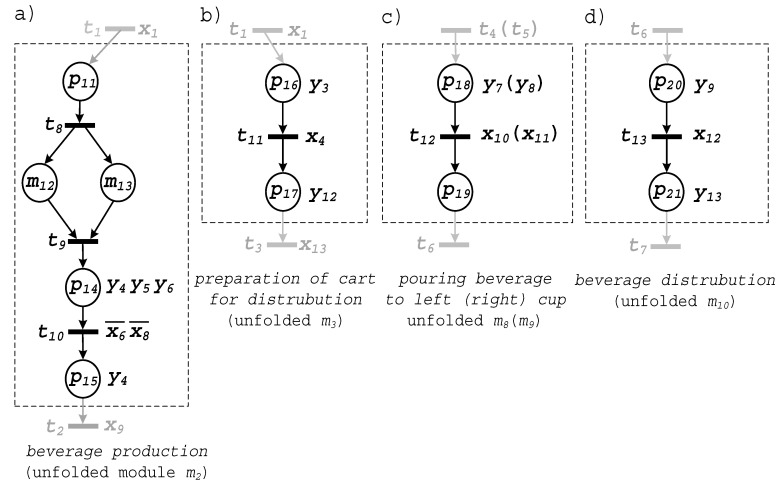
Unfolded top module (first “unfolding” level): (**a**) *m*_2_, (**b**) *m*_3_, (**c**) *m*_8_ (*m*_9_), (**d**) *m*_10._

The first one ($m_{2}$) refers to the production of the beverage. Figure 6a presents the unfolded content of this module. It contains five places and three transitions. Note that places $m_{12}$ and $m_{13}$ are the macroplaces that are going to be unfolded at the subsequent step of the proposed modelling methodology. Operations within these two modules are executed concurrently: preparation of the ingredients in the first ($m_{12}$) and in the second ($m_{13}$) container, respectively. When the ingredients are ready (transition $t_{8}$), the output valves of both containers are opened ($y_{5}$ and $y_{6}$, respectively), pouring the beverage into the third container. This process goes on until both containers are empty (which is signalized by sensor $x_{6}$ for Container 1, and by $x_{8}$ for Container 2). Both ingredients are mixed ($y_{4}$) until the remaining time elapses (signal $x_{9}$). The production process is finished and the beverage is ready for the distribution.

Unfolded macroplace $m_{3}$ is presented in Figure 6b. It contains two places and one transition. Place $p_{16}$ is associated with the placement of cups on the distribution cart (operation $y_{3}$). When both cups are located on the cart (signalized by sensor $x_{4}$), the cart is moved to the left position ($y_{12}$).

Modules $m_{12}$ and $m_{13}$ respond to the pouring of the beverage into the left and right cups of the cart, respectively. Since their functionalities are similar, they are illustrated by the same figure (Figure 6c). Initially, the valve $y_{7}$ (or $y_{8}$ for module $m_{13}$) is opened. The left (right) cup is filled up until reaching sensor $x_{10}$ ($x_{11}$). Note that such a representation of the similar modules is an additional advantage of the proposed modelling technique because it is possible to prepare a common description for various modules. This approach is similar to applying the functions and procedures used in programming languages, or instances in hardware description languages.

Finally, unfolded module $m_{10}$ consists of two places, executing operations related to the distribution of the beverage. The cart transfers the produced beverage. Once the cart reaches the rightmost position (notified by sensor $x_{12}$), the cups are taken from the cart ($y_{13}$). The specification of this module is shown in Figure 6d.

**Figure 7:**
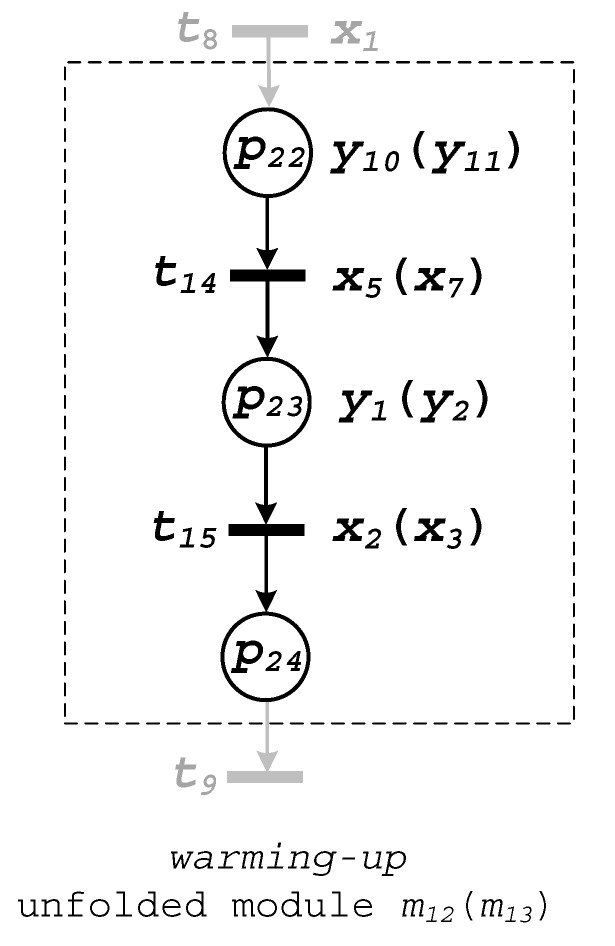
Unfolded module m_12_ (m_13_).

Now all the modules of the top-level are unfolded. Subsequently, in further steps, the nested modules are unfolded. There are only two of them, both located in module $m_{2}$. Namely, modules $m_{12}$ and $m_{13}$ have to be processed. Both modules have similar functionalities that refer to the production of the beverage in the first (or second) container. Therefore, they are presented commonly, in Figure 7. The valve of the container (output $y_{10}$ for Container 1, and $y_{11}$ for Container 2) is opened, and the container is filled up with the liquid ingredients. This process goes on, until the proper level is achieved (signalized by sensor $x_{5}$ for Container 1 and sensor $x_{7}$ for Container 2). Next, the ingredients are warmed up (actions $y_{1}$ and $y_{2}$) to achieve the proper temperature, which is signalized by sensors $x_{2}$ and $x_{3}$, respectively.

Unfolding of macroplaces $m_{12}$ and $m_{13}$ finishes this stage of the modelling. All the modules at every level are unfolded, and the system can be formally verified.

### 6.3. Formal Verification of Unfolded Modules

According to the proposed bottom-up verification approach, firstly, the bottom-level modules are formally verified, then the modules at subsequent upper levels are examined, until the top-level is reached. In the considered example, there are three levels of hierarchy with the specified modules, namely: (I) $m_{3}$, $m_{8}$,$\;m_{9}$,$\;m_{10}$,$\;m_{12}$,$\;m_{13}$, (II) $m_{2}$, and (III) the top-level.

Each module before the formal verification is transformed by connecting its output place with its input place, e.g., in module $m_{12}$, place $p_{24}$ is merged with place $p_{22}$. This operation permits the checking of the liveness property of a subnet.

Next, the modified modules are formally verified using a model checking technique. In the experiments, we have used the nuXmv tool, as an up-to-date model checker, and the m2vs tool (“model to verification and synthesis”), as an implemented tool for the model transformations. Additionally, the idea of a rule-based logical model has been applied (described in detail in [44] and [47]). It simplifies the verification process, as far as the verifiable models are generated automatically and are ready to be imported into the model checker tool. The only thing the designer has to do is to describe a net (or a subnet) in text form (the verifiable model is then generated) and to prepare the list of requirements. These are usually delivered in the form of the temporal logic formulas, but it is also possible (when needed, e.g., in the case of a user-centred design where the customer is involved in the development process) to simplify the requirement definition process by using the alternate forms, such as a user-friendly Scratch-based definition [71].

According to the bottom-up approach, formal verification starts at the lowest level of system hierarchy. Subsequently, it is performed at various hierarchy levels, each time taking into account the properties of the considered part of the system, and so, each looped first-level module is described as a rule-based logical model, then transformed into a verifiable model and formally verified with the list of requirements. Having checked all bottom-level modules, the second-level hierarchy modules are checked (in this case it is only the $m_{2}$ module) in the same way as the first-level modules. What should be noted is the fact that when considering the higher-level modules, the rule-based logical model directly reflects their structure, e.g., in module $m_{2}$ there are three places $p_{11}$, $p_{14}$, $p_{15}$ and two macroplaces $m_{12}$ and $m_{13}$ (and, as in the other modules from lower hierarchy levels, place $p_{15}$ is joined with place $p_{11}$ for analysis purposes). This permits focussing on the particular functionality at one time and additionally eliminates the state-explosion in the model checking process. The third level of hierarchy in the considered case study is the top module, and its formal verification is described in the following subsection.

The requirements to be checked in the case study concern the subnets’ structures (with initial marking) and behaviours. They have been defined using computation tree logic (CTL). Sample properties for checking reachable states are specified in the nuXmv format as “CTLSPEC EF (p15)” (for the unfolded module $m_{2}$). Behavioural properties are of great importance too, as they refer to input and output signals and their correlation with each other and with the net structure. For example, defined requirement “CTLSPEC EF (y4 and y5 and y6)” (for module $m_{2}$) checks whether a state is reachable where the three output signals (mixing and opened output valves of both containers) are active at the same time. Indeed, it turns out to be reachable, which is a desired system behaviour.

### 6.4. Formal Verification of the Top Module

Finally, the top level net is verified. Its rule-based logical model directly describes the net shown in Figure 5. Similar to the previous stages of formal verification, the rule-based logical model is automatically transformed into a verifiable model, a list of requirements is defined (with CTL) and the two elements are compared with each other. Here, the global properties of the system become more important, especially when considering the desired behaviour of the control part. Sample behavioural properties are, e.g., that it should never be the case that ingredients are being mixed (active output signal $y_{4}$) while a valve for pouring the beverage is open (active output signals $y_{7}$ or $y_{8}$), or that any valve for pouring the beverage to the cup on the cart is opened (active output signals $y_{7}$ or $y_{8}$) while the cart is moving back (active output signal $y_{9}$).

Let us emphasize that, in the proposed modelling flow (as shown in the case study), formal verification with model checking strongly focuses on behavioural requirements, as it is very important to check the correlation between input and output signals. Additionally, it is essential to confirm that “something bad will never happen” (these are called safety properties, e.g., two signals can never be active at the same time) and that “something good will eventually happen” (these are called liveness properties, e.g., it is always possible to reach a state where a particular signal is active). It should be noted that safety and liveness properties do not directly refer to the definitions of safeness and liveness in Petri nets (Definitions 7 and 8), as far as they are wider. Liveness and safeness in the sense of Definitions 7 and 8 can be checked via the methods of Petri net analysis, as it was mentioned in Section 5, and the liveness and safeness properties in a wider sense can be described by means of temporal logic formulae and verified using model checking.

If all the requirements are met, the development process may proceed to further stages leading to the final implementation. In the reverse case, the error source should be localized and eliminated, and the verification should be performed again until all requirements are satisfied.

## 7. Experimental Verification of the Determinism of a System Modelled According to the Proposed Method

The proposed modelling technique has been verified experimentally. In particular, the system shown in the previous section was modelled, verified and simulated with the use of the Platform Independent Petri Net Editor (PIPE) 2 (ver. 4.3.0). This is a Java-based tool that allows the construction and analysis of Petri net-based models [68]. Besides the standard functionalities, PIPE provides extension by additional pluggable analysis modules, such as the verification of liveness, safeness, reachability graph computation, place invariant computation, etc. In our investigations, the tool was used to analyse the behaviour of the system (especially in regard to determinism) and to verify the crucial properties of the Petri net (liveness, safeness). Let us briefly present the methodology of the experimental set-up.

According to the proposed methodology, the verification is executed with the bottom-up technique. Initially, the bottom modules of the system $m_{12}$ and $m_{13}$ (Figure 7) were examined. The verification was executed with the set of tools and extensions available within the PIPE tool. In particular, the following properties were examined:

- State space analysis: both modules (after connecting of terminal and initial places) are safe and live;
- Reachability/coverability graph: there are three reachable states (markings) in each module;
- Classification: both modules belong to the state machine class [45].

The most important information from the above results refers to the liveness and safeness. It means that both macroplaces meet the criteria of MCIPN specified by Definition 14. 

The deterministic behaviour of the macroplaces was examined by the simulation. In particular, the animation mode of the PIPE tool was applied. Such an approach permits the detailed analysis of the system in regard to the transition firings. In the presented example, all the possible options were checked. It means that each possible state of the module was carefully examined in regard to the deterministic behaviour of the system. The analysis shows that both modules are strongly deterministic, since they fulfil all the restrictions specified by Theorem 2.

Similarly to the above procedure, the modules at the upper level (Figure 6) are analysed. The verification provided the following results:

- Modules $m_{2},m_{3},\;m_{8},m_{9},\;m_{10}$ are (after joining of terminal and initial places) live and safe, thus they fulfil definition of MCIPN.
- Modules $m_{3},\;m_{8},m_{9},\;m_{10}$ are strongly deterministic (according to Theorem 2).
- Module $m_{2}$ is weakly deterministic (according to Theorem 1 and Theorem 3).

Let us discuss the deterministic aspects of module $m_{2}$. Note that the structure of this module is strongly deterministic, since all the conditions given by Theorem 2 are fulfilled. However, it contains internal macroplaces $m_{12}$ and $m_{13}$ which are executed concurrently and violate assumptions of Theorem 2. Nevertheless, all restrictions of Theorem 1 are met, thus the module is weakly deterministic (which also follows from Theorem 3).

**Figure 8:**
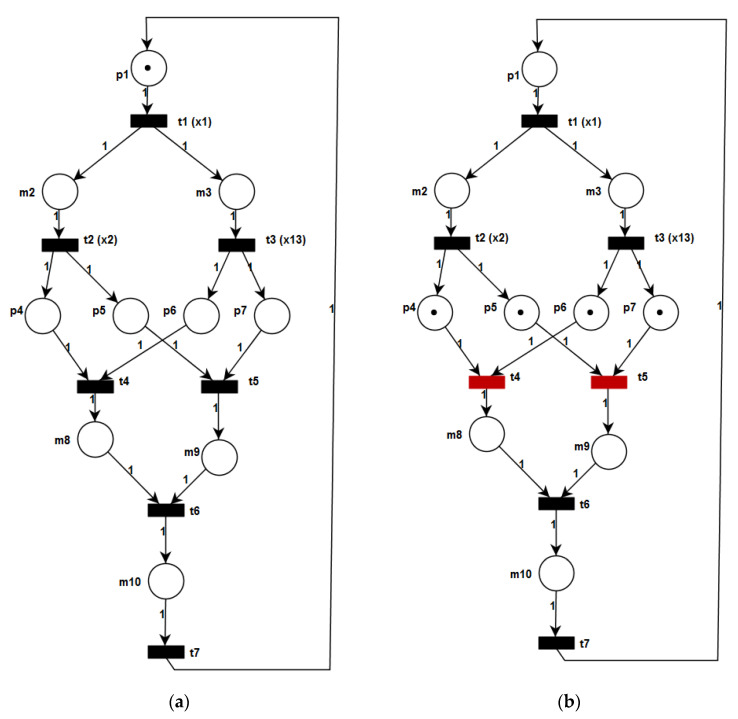
Model of the top module (**a**) and its simulation (**b**) in PIPE.

Finally, the top module is analysed. Let us describe the modelling, verification and analysis of this module in more detail, since several interesting properties can be noticed. Figure 8a shows the top module specified with the PIPE tool. The verification of the top module provides the following results:

- Petri net state space analysis: the net is safe and live;
- Reachability/coverability graph: there are eight reachable states;
- Classification: the net belongs to the marked graph class [45].

From the above properties it can be noticed that the top module is live and safe. Therefore, all the properties of HCIPN (Definition 15) are fulfilled. 

The simulation of the module shows that the top module is weakly deterministic. In order to demonstrate the analysis technique more closely, let us illustrate by example. Figure 8b presents a state of the module, where four places are simultaneously marked: $p_{4}$, $p_{5},$ $p_{6},\;$and $p_{7}$. It refers to the situation where the beverage production has already been completed, and the cart is already placed at the requested position (sensor $x_{13}$). Note that two transitions are enabled in this marking: $t_{4}$ and $t_{5}$. Clearly, it is not possible to determine the order of transition firings. This means that according to Theorem 2 the top module is not strongly deterministic. The remaining states of the top module were examined in a similar way which showed that this module is weakly deterministic.

Summarizing the experimental verification of the system let us emphasize that the presented system is live and safe. Furthermore, it contains strongly deterministic MCIPNs (at the bottom and intermediate level of the hierarchy), but the system as a whole is weakly deterministic (because of the top module and macroplace $m_{2}$). 

It can be seen on the example of the considered case study that the proposed approach, due to using hierarchy both at the steps of modelling and verification, reduces the number of states (markings) which have to be explored. The complete exploration of reachable states of all the modules (taking into account the joining of the initial and terminal places of the modules as it was described above) covers 16 markings. A similar analysis of the whole system, without using hierarchical decomposition, requires an exploration of 22 markings. Of course, for more complicated systems the difference is much greater.

## 8. Conclusions

This work is focused on the deterministic aspects of the control part of a CPS modelled by the control interpreted Petri nets. Novel theorems and definitions oriented toward the deterministic modelling of such systems have been introduced. Moreover, a modelling methodology of a deterministic system specified by a CIPN has been proposed. The proposed ideas have been explained using a case study example of a real-life cyber-physical system and verified experimentally. 

The main benefits of the proposed approach can be summarized as follows:

1. The way of design provides a description which is easy to understand and to handle, it reduces the probability of the mistakes.
2. The way of verification simplifies the assessment of the properties which have to be analysed and makes localization easy, as well as the correction of the inconsistences.
3. Focussing on the determinism guarantees a predictable behaviour of the system, i.e. its reaction on any specific combination of input values in one possible way, at the same time taking advantage of parallelism.

The application of interpreted Petri nets utilizes their unique features, especially including the graphical modelling of concurrency and possibility of formal verification of the system. Furthermore, two different kinds of determinism are proposed: weak and strong determinism. Finally, the idea is supported by the adequate modelling methodology. 

On the other hand, the proposed techniques have several limitations. First, the presented solution heavily utilizes the Petri net theory, which may be difficult for the designers that are not familiar with this modelling methodology. Furthermore, modelling the system according to the presented methodology enforces several restrictions related to weak and strong determinism.

Future plans include the enhancement of the presented modelling methodology by further steps, especially focussing on the implementation aspects. In particular, it is planned to propose the deterministic design methodology for CPS realized as distributed and integrated systems. Moreover, analyses of the timing aspects of the systems modelled with the proposed methodology are going to be considered (taking into account logical and physical time).
